# Potential Survival Benefit of Neoadjuvant Docetaxel, Cisplatin and 5‐Fluorouracil Therapy in Patients With Esophageal Squamous Cell Carcinoma With Multiple Lymph Node Metastases: A Single‐Institute Propensity Score Analysis

**DOI:** 10.1002/ags3.70224

**Published:** 2026-04-19

**Authors:** Eiji Higaki, Tetsuya Abe, Hironori Fujieda, Shigenori Kadowaki, Tsutomu Tanaka, Masahiro Tajika, Koji Komori, Seiji Ito, Isao Oze, Kei Muro

**Affiliations:** ^1^ Department of Gastroenterological Surgery Aichi Cancer Center Hospital Nagoya Aichi Japan; ^2^ Department of Clinical Oncology Aichi Cancer Center Hospital Nagoya Aichi Japan; ^3^ Department of Endoscopy Aichi Cancer Center Hospital Nagoya Aichi Japan; ^4^ Division of Cancer Information and Control Aichi Cancer Center Research Institute Nagoya Aichi Japan

**Keywords:** 5‐fluorouracil, cisplatin, docetaxel, esophageal squamous cell carcinoma, lymph node metastasis, neoadjuvant therapy, propensity score analysis

## Abstract

**Background:**

Neoadjuvant chemotherapy with fluorouracil, cisplatin, and docetaxel (NAC‐DCF) followed by surgery is the current standard of care for resectable advanced esophageal squamous cell cancer (ESCC) in Japan based on the JCOG1109 trial. Although NAC‐DCF improves survival, it also increases the risk of febrile neutropenia. Therefore, it is essential to identify tumor factors that predict the greatest benefit from NAC‐DCF over conventional fluorouracil and cisplatin chemotherapy (NAC‐CF) to optimize patient selection.

**Methods:**

We retrospectively analyzed patients with resectable advanced ESCC who received either NAC‐CF or NAC‐DCF at our institution between 2006 and 2019. Propensity score‐based inverse probability weighting (IPW) and multivariable adjusted analyses were used to consider baseline differences and compare overall survival (OS) stratified by cTNM factors between groups.

**Results:**

A total of 408 patients received NAC‐CF, while 218 received NAC‐DCF. NAC‐DCF was applied to more advanced patients, with 86% having cT3‐4a and 52% having cN2‐3 (both *p* < 0.001). After IPW, the hazard ratio (HR) for OS in the NAC‐DCF group was 0.74 [95% confidence interval (CI), 0.55–0.98]. Subgroup analysis showed that patients with cN2‐3 had a significant survival benefit with NAC‐DCF (HR 0.51; 95% CI, 0.34–0.76) in the IPW analysis, whereas no clear association was observed in those with cN0–1 (HR 0.93; 95% CI, 0.62–1.34), with a nominally significant interaction by clinical N stage (*p* = 0.039).

**Conclusion:**

Neoadjuvant chemotherapy with fluorouracil, cisplatin, and docetaxel (NAC‐DCF) was associated with improved survival, with a potential benefit in patients with advanced nodal involvement; however, these findings should be interpreted as hypothesis‐generating given the retrospective design.

## Graphical abstract

Although neoadjuvant chemotherapy with fluorouracil, cisplatin, and docetaxel (NAC‐DCF) is the current standard neoadjuvant regimen for esophageal squamous cell carcinoma, its substantial toxicity underscores the need to identify patients who derive the greatest benefit. A retrospective comparison was made of NAC‐DCF (*n* = 218) with conventional fluorouracil and cisplatin chemotherapy (NAC‐CF) (*n* = 408) using multivariable‐adjusted analyses and an inverse probability weighting (IPW) model. NAC‐DCF was associated with improved overall survival in patients with cN2–3 disease, whereas no clear association was observed in those with cN0–1 disease. These findings suggest that clinical N stage may help refine patient selection for intensive neoadjuvant chemotherapy.

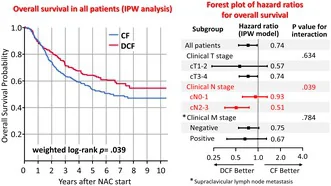

## 1 Introduction

In Europe and the United States, neoadjuvant chemoradiotherapy (CRT) followed by surgery has been established as the standard treatment for resectable advanced esophageal squamous cell carcinoma (ESCC) [1, 2]. Meanwhile, in recent years, triplet or more neoadjuvant chemotherapy (NAC) has gained increasing recognition in these regions, particularly for esophageal adenocarcinoma, with efficacy supported by the results of the Neo‐AEGIS [3] and ESOPEC [4] trials. In Japan, the standard perioperative treatment for resectable advanced ESCC is neoadjuvant chemotherapy followed by surgery. The doublet regimen of fluorouracil and cisplatin (CF) had been the standard NAC regimen based on the JCOG9907 trial [5], and recently, the JCOG1109 phase III prospective randomized trial demonstrated that a triplet NAC regimen comprising fluorouracil, cisplatin, and docetaxel (DCF) improved 3 year survival rates by approximately 10% compared with CF [6], establishing NAC‐DCF as the standard treatment.

While triplet therapy improves prognosis, the DCF regimen is associated with a higher incidence of severe grade 3 or 4 adverse events, including neutropenia, hypernatremia, and anorexia. Notably, febrile neutropenia (FN) occurred in 16% of patients treated with the DCF regimen, compared with 1% in those receiving the CF regimen, and the incidence of FN reached 25%–35% in patients treated with the NAC‐DCF regimen in our institute [7, 8].

Therefore, in clinical practice, the conventional doublet CF regimen, which is associated with fewer adverse events, remains a treatment option for elderly patients or those with severe comorbidities [9]. However, from an oncological perspective, DCF therapy has shown favorable outcomes in more advanced ESCC, such as in patients with locally advanced unresectable disease [7], and its application may be inevitable in more advanced stages. It is thus crucial to identify clearly the oncological factors that make the NAC‐DCF regimen particularly suitable for patients within the NAC‐eligible population.

In the post hoc analysis of JCOG1109, no clearly defined patient subgroup that derived the greatest benefit could be identified. Given the multicenter design of JCOG1109, which involved 44 institutions, together with the inherent challenges in accurately diagnosing lymph node metastasis, some degree of inter‐institutional variation is unavoidable. While the evidence generated by JCOG1109 remains definitive, evaluating outcomes within a high‐volume, single‐institution setting—where diagnostic criteria, treatment strategies, and perioperative management are applied consistently—may reduce heterogeneity and provide clinically relevant real‐world evidence. Such an approach can complement data from randomized trials and help refine the clinical interpretation of outcomes with NAC‐DCF.

This study aimed to explore which patients may derive the greatest survival benefit from NAC‐DCF compared with NAC‐CF in a large single‐institution cohort.

## 2 Methods

### 2.1 Patients

Consecutive patients aged ≤ 75 years who underwent NAC for thoracic ESCC between 2006 and 2019 were initially identified from a prospectively maintained database at Aichi Cancer Center Hospital. Eligible NAC regimens included either CF or DCF. The inclusion criteria for tumor stage were based on clinical diagnosis as cT1N1–3 or cT2‐4a N0‐3, (according to the 8th edition of the Union for International Cancer Control staging system). cM1 were included only if the metastasis was confined to the supraclavicular lymph nodes (SCLNs). Patients with tumors deemed unresectable at diagnosis were excluded. All study patients submitted a general consent form at the time of their initial diagnosis and agreed to participate in the study. The review board of the Aichi Cancer Center Hospital approved this study (Approval No. ACC 2021–1‐043).

### 2.2 Clinical Staging

Pre‐treatment clinical staging was performed in all patients using endoscopy and computed tomography (CT) with a slice thickness of less than 5 mm. Positron‐emission tomography (PET/CT) was performed in approximately 80% of patients as part of routine staging practice. Fluorodeoxyglucose (FDG) PET/CT scans were performed using a Siemens Biograph 40 TruePoint w/TrueV (Siemens Healthineers). Patients fasted for ≥ 6 h before receiving ^18^F‐FDG (3.7 MBq/kg), and imaging was initiated approximately 90 min later [Correction added on 2 May 2026, after first online publication: The citation of Reference 10 in Section 2.2 | Clinical Staging has been removed, and the reference list has been renumbered accordingly.]. Images were obtained from the vertex to the mid‐thigh with CT‐based attenuation correction and reconstructed using standard iterative algorithms. Findings were interpreted in SUV mode for semiquantitative evaluation using syngo. via software (Siemens Healthineers).

The diagnosis of lymph node (LN) metastasis required a short axis diameter of at least 5 mm in the region of the recurrent nerve, paraesophagus, with confirmation based on additional morphological features, including a round shape, internal necrosis, irregular margins, and marked contrast enhancement. LNs located at the tracheal bifurcation or in the abdomen were considered potentially metastatic if their short‐axis diameter exceeded 1 cm. LNs in the pulmonary hilar and pre‐tracheal regions were generally not deemed metastatic, as they often exhibit inflammatory enlargement. However, they were considered metastatic if they demonstrated the aforementioned morphological characteristics. In patients of diagnostic uncertainty on the CT, PET/CT was used for further evaluation. Importantly, even if FDG uptake was noted on PET/CT, it was not considered metastatic if no suspicious LNs were identified on CT. The final clinical diagnosis was determined through a multidisciplinary team conference involving esophageal surgeons, endoscopists, radiologists, and medical oncologists. Clinical (c) and pathological (p) staging was classified based on the TNM classification of the International Union Against Cancer, 8th Edition.

### 2.3 Neoadjuvant Chemotherapy (NAC)

Treatment strategies, including NAC regimens, were determined through a multidisciplinary team conference based on a comprehensive evaluation of resectability, nodal status, patient performance status, comorbidities, and organ function. NAC‐CF remained a standard treatment throughout the study period, while DCF was also widely adopted, and treatment selection was based on clinical assessment rather than treatment era.

The standard CF regimen comprised two courses, with each course consisting of cisplatin (80 mg/m^2^/day on day 1) and 5‐fluorouracil (800 mg/m^2^/day on days 1 to 5) [5]. The interval between each course was 3 weeks. The standard DCF regimen comprised 2–3 courses, with each course consisting of docetaxel (60–70 mg/m^2^ per day on day 1), cisplatin (60–80 mg/m^2^ per day on day 1) and 5‐fluorouracil (750–800 mg/m^2^ per day on days 1 to 5). The interval between each course was 3–4 weeks; details of the NAC‐DCF therapy are outlined in our previous study [10].

### 2.4 Surgical Procedure

A radical subtotal esophagectomy was performed with regional LN dissection, including both cervical and thoracic paraoesophageal, paratracheal, subcarinal, mediastinal and perigastric nodes. In addition to the above LNs, in principle, a three‐field LN dissection, including the SCLNs, was also conducted but was selectively omitted based on the patient's overall condition and tumor status. The details of our surgical technique have been reported previously [11].

### 2.5 Adjuvant Treatment

For patients who received NAC, no postoperative adjuvant treatment was administered in accordance with the Japanese esophageal cancer practice guidelines.

### 2.6 Outcomes

Overall survival (OS) was measured from the start of NAC to death or censoring at the last follow‐up. A stratified analysis based on cTMN factors was performed to identify the tumor characteristics most favorable for NAC‐DCF therapy. Compliance with NAC was assessed by examining the proportion of patients who failed to undergo the planned surgery for reasons other than unresectable disease. Surgical outcomes were evaluated based on intraoperative findings, postoperative complications, pathological results, R0 resection rate, and NAC response. Relapse‐free survival (RFS) was defined from surgery to recurrence or censoring. Recurrence patterns were also analyzed.

### 2.7 Statistical Analysis

Continuous variables are presented as medians (interquartile ranges [IQRs]), and categorical variables are presented as numbers (percentages). Differences between the 2 groups were assessed using the chi‐square test or Fisher's exact test for categorical variables and the Mann–Whitney U test for continuous variables, when appropriate. All reported *p* values are two‐sided; *p* < 0.05 was considered significant.

To minimize the impact of potential confounding effects in this observational study, we used weighted propensity score analysis to adjust for significant differences in patient characteristics. Propensity scores (PS) were calculated as the conditional probability of being assigned to either NAC‐CF or NAC‐DCF, using a logistic regression model that incorporated preoperative variables, including key confounding factors related to survival: age, gender, American Society of Anesthesiologists physical status (ASA‐PS), body mass index (BMI), treatment period, cT and cN stages and SCLN metastasis. Each patient was assigned a weight based on the inverse probability of being allocated to either group, with the aim of balancing observable characteristics.

Crude and inverse probability weighting (IPW) survival rates were computed using the Kaplan–Meier method. The Cox proportional hazards model was employed to estimate the treatment effect, with adjustments made for the prognostic factors of survival previously described. These adjustments were made using the multivariable adjusted model and the IPW model.

All statistical analyses were performed with the statistical software packages STATA ver.16.1 (STATA Corp. College Station, TX, USA).

## 3 Results

### 3.1 Patient Characteristics

During the study period, 626 patients fulfilled the inclusion criteria. Of these, 408 patients (65.2%) were treated with the NAC‐CF regimen (the CF group), and 218 (34.8%) were treated with the NAC‐DCF regimen (the DCF group). A comparison of baseline characteristics between the two regimens revealed that the DCF group had more advanced cT, cN stages, as well as a higher prevalence of SCLN metastases, compared with the CF group (Table 1).

**TABLE 1:** Pre‐treatment baseline characteristics.

Variable	CF (*n* = 408)		DCF (*n* = 218)		*p* Value
Year of NAC start ≥ 2014	200	(49.0%)	115	(52.8%)	0.374
Age, median (IQR), yr	65	(60–70)	64	(58–69)	0.028
Male, *n* (%)	337	(82.6%)	183	(83.9%)	0.669
BMI median (IQR), kg/m^2^	21.6	(19.4–23.4)	21.4	(18.8–23.1)	0.100
ASA‐PS, *n* (%)					0.435
1	140	(34.3%)	83	(38.1%)	
2	225	(55.2%)	118	(54.1%)	
3	43	(10.5%)	17	(7.8%)	
Diabetes mellitus, *n* (%)	47	(11.5%)	20	(9.2%)	0.366
Cardiovascular disease, *n* (%)	172	(42.2%)	85	(39.0%)	0.443
FEV1.0%, median (IQR), %	77.0	(72.4–81.0)	76.5	(72.1–81.4)	0.804
Brinkman index, median (IQR)	660	(350–1000)	600	(350–960)	0.455
Tumor location					0.341
Proximal third	55	(13.5%)	39	(17.9%)	
Middle third	214	(52.5%)	109	(50.0%)	
Distal third	139	(34.1%)	70	(32.1%)	
Clinical T stage^a^					< 0.001
1	98	(24.0%)	12	(5.5%)	
2	74	(18.1%)	18	(8.3%)	
3	234	(57.4%)	182	(83.5%)	
4a	2	(0.5%)	6	(2.8%)	
Clinical N stage^a^					< 0.001
0	58	(14.2%)	17	(7.8%)	
1	233	(57.1%)	88	(40.4%)	
2	109	(26.7%)	99	(45.4%)	
3	8	(2.0%)	14	(6.4%)	
Supraclavicular lymph node metastasis	21	(5.2%)	44	(20.2%)	< 0.001

Abbreviations: ASA‐PS, American Society of Anesthesiologists physical status; BMI, body mass index; CF, fluorouracil and cisplatin; DCF, fluorouracil, cisplatin, and docetaxel; IQR, interquartile range; NAC, neoadjuvant chemotherapy.

^a^ TNM classification of International Union Against Cancer (UICC) 8th edition.

### 3.2 Treatment Compliance

**FIGURE 1:**
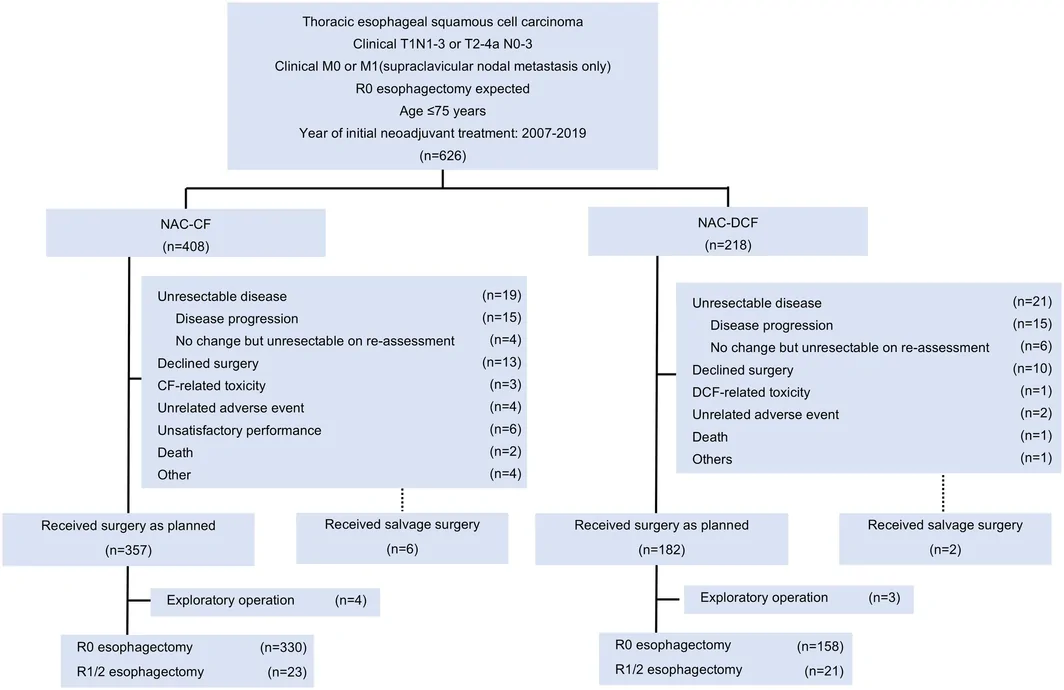
Flowchart of patient enrolment: NAC‐CF is a doublet neoadjuvant chemotherapy (NAC) regimen consisting of fluorouracil and cisplatin. NAC‐DCF is a triplet NAC regimen consisting of fluorouracil, cisplatin, and docetaxel.

A flowchart of the treatment process for all patients is shown in Figure 1. R0 resection was achieved in 330 of 408 patients in the CF group and 158 of 218 patients in the DCF group (80.9% vs. 72.5%, respectively; *p* = 0.016). While patients in the DCF group with advanced clinical stage were less likely to achieve R0 resection compared with those in the CF group, mainly due to unresectable disease, failure to undergo planned surgery for reasons other than unresectable disease occurred in 32 patients in the CF group and 15 patients in the DCF group, with similar incidence rates (7.8% versus 6.9%, respectively; *p* = 0.663). The most common reason for not having surgery, aside from unresectable disease, was patient refusal, while drug toxicity rarely prevented surgery in either group.

### 3.3 Surgical Outcomes and Tumor Pathology

Among patients who underwent esophagectomy as planned, the surgical information was compared between the two groups (Table 2). The duration from NAC start to surgery was longer in the DCF group, as their default regimen was designed to be one course more than that in the CF group. The DCF group had a greater amount of intraoperative blood loss due to advanced tumor stage, but had a significantly lower incidence of postoperative complications, such as pneumonia, anastomotic leakage and recurrent laryngeal nerve palsy.

**TABLE 2:** Surgical outcomes and tumor pathology among patients undergoing planned esophagectomy.

Variable	CF (*n* = 353)		DCF (*n* = 179)		*p* Value
Duration from NAC start to surgery, median (IQR), days	64	(57–74)	77	(68–90)	< 0.001
Operative procedure, *n* (%)					0.114
Transthoracic esophagectomy	177	(50.1%)	103	(57.5%)	
Thoracoscopic esophagectomy	171	(48.4%)	76	(42.5%)	
Transhiatal esophagectomy	5	(1.4%)	0	(0.0%)	
Three‐field lymph node dissection, *n* (%)	321	(90.9%)	170	(95.0%)	0.121
Conduit for reconstruction, *n* (%)					0.107
Stomach	320	(92.5%)	172	(96.1%)	.
Pedicled jejunum	26	(7.5%)	7	(3.9%)	
Intraoperative findings					
Operation time, median (IQR), min	438	(395–505)	451	(407–512)	0.157
Blood loss, median (IQR), mL	210	(110–350)	270	(150–450)	0.004
Postoperative complications, *n* (%)					
Overall morbidity	230	(65.3%)	109	(60.9%)	0.313
Pneumonia	86	(24.4%)	31	(17.3%)	0.062
Anastomotic leakage	47	(13.4%)	12	(6.7%)	0.021
Recurrent laryngeal nerve palsy	86	(24.4%)	28	(15.8%)	0.023
Surgical site infection	39	(11.1%)	22	(12.3%)	0.679
ARDS	10	(2.8%)	4	(2.2%)	0.680
Length of postoperative hospital stay, median (IQR), days	22	(17–32)	21	(16–27)	0.067
30‐day mortality, *n* (%)	0	(0.0%)	0	(0.0%)	—
90‐day mortality, *n* (%)	1	(0.3%)	0	(0.0%)	0.477
Pathological T stage^a^					< 0.001
0	24	(6.8%)	30	(16.8%)	
1	143	(40.5%)	47	(26.3%)	
2	52	(14.7%)	26	(14.5%)	
3	126	(35.7%)	67	(37.4%)	
4	8	(2.3%)	9	(5.0%)	
Pathological N stage^a^					0.936
0	126	(35.7%)	67	(37.4%)	
1	120	(34.0%)	58	(32.4%)	
2	65	(18.4%)	35	(19.6%)	
3	42	(11.9%)	19	(10.6%)	
Total number of lymph node metastases	1	(0–3)	1	(0–3)	0.905
Supraclavicular lymph node metastasis	30	(8.5%)	26	(14.5%)	0.046
Residual tumor					0.099
R0	330	(93.5%)	158	(88.3%)	
R1	20	(5.7%)	18	(10.1%)	
R2	3	(0.9%)	3	(1.7%)	
Histological NAC response on primary site (%)^b^					< 0.001
Grade 0–1a	114	(32.3%)	31	(17.3%)	
Grade 1b	123	(34.8%)	46	(25.7%)	
Grade 2	89	(25.2%)	72	(40.2%)	
Grade 3 (No residual tumor)	242	(6.8%)	30	(16.8%)	
Unknown	3	(0.9%)	0	(0.0%)	

Abbreviations: ARDS, acute respiratory distress syndrome; CF, fluorouracil and cisplatin; DCF, fluorouracil, cisplatin, and docetaxel; IQR, interquartile range; NAC, neoadjuvant chemotherapy.

^a^ TNM classification of the International Union Against Cancer (UICC) 8th edition.

^b^ Histopathological response was evaluated according to the Japanese Classification of Esophageal Cancer, 11th edition (Grade 0: no area of degeneration; Grade 1a: viable tumor cells accounting for two‐thirds or more of tumor tissue; Grade 1b: viable tumor cells accounting for between a third and two‐thirds of tumor tissue; Grade 2: viable tumor cells accounting for less than a third of tumor tissue; and Grade 3: no viable tumor cells).

Among patients who underwent esophagectomy as planned, the pathology of the resected specimens was examined (Table 2). Downstaging from clinical to pathological stage was more pronounced in the DCF group compared with the CF group. As a result, the differences in cT and cN stages observed between groups were no longer evident at the pathological stage, and the DCF group demonstrated a higher rate of patients with no residual tumor at the primary site (6.8% versus 16.8%, respectively; *p* < 0.001).

### 3.4 Survival Analysis

**FIGURE 2:**
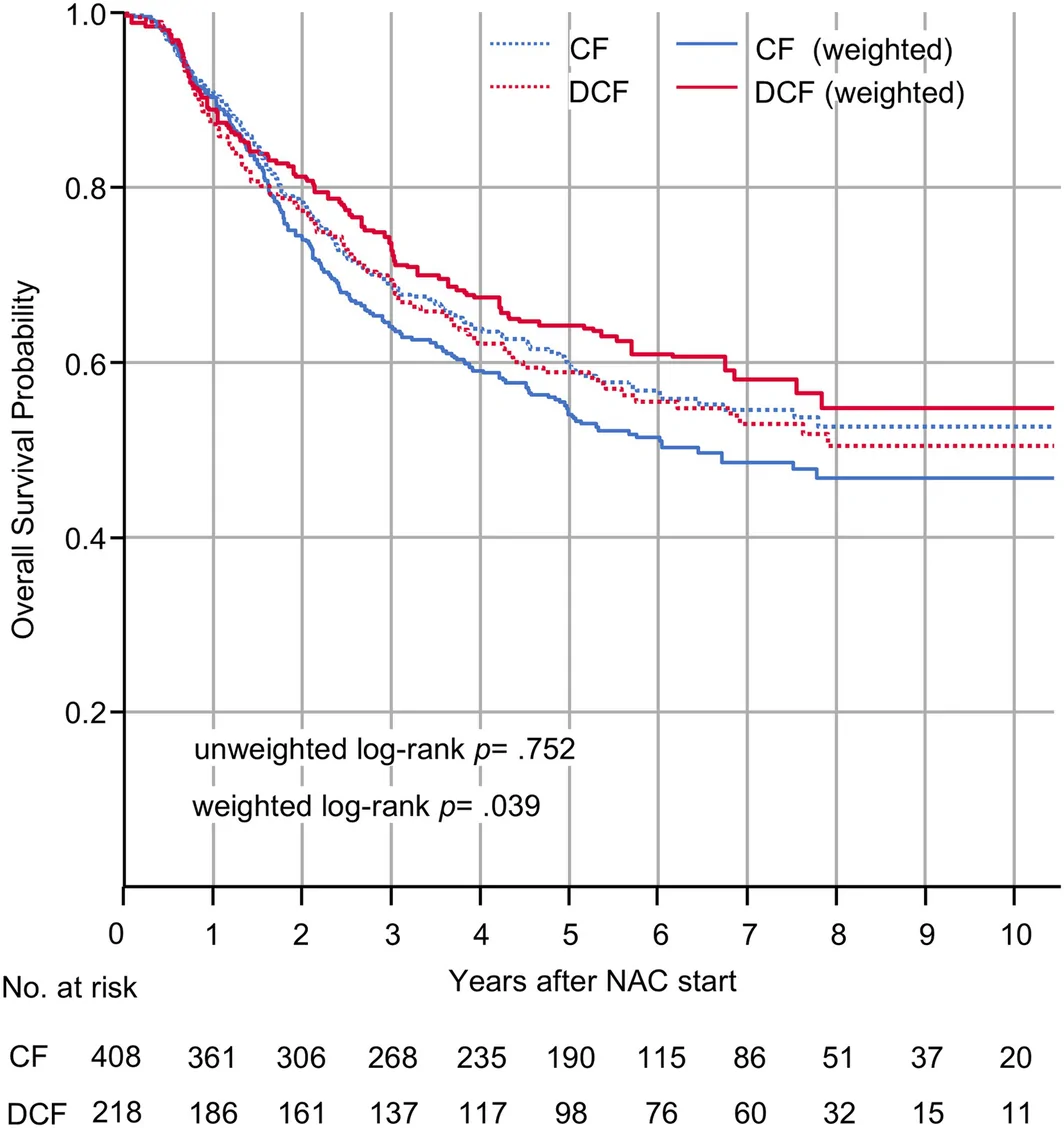
Kaplan–Meier estimates for crude and inverse probability weighting overall survival curves in all patients between the NAC‐CF and NAC‐DCF groups.

Survival analysis included all patients who started NAC, and the median follow‐up for surviving patients was 70.1 months (IQR, 58.3–93.6). The crude survival curves for the CF and DCF groups overlapped, showing no differences between groups (Figure 2; dashed line). This analysis was repeated after IPW. The density distribution of propensity scores in both groups is shown in Figure S1a. The IPW effectively balanced the key covariates across the cohort, as evidenced by the improvement in the standardized mean difference (SMD) (Figure S1b). The weighted OS rates at 1, 3, and 5 years after NAC start were 89.6%, 64.5% and 54.5%, respectively, in the CF group, as compared with 89.2%, 74.7% and 64.6%, respectively in the DCF group (Figure 2; solid line, *p* = 0.039 by weighted log‐rank test), indicating improved OS in the DCF group (weighted hazard ratio [wHR], 0.74; 95% confidence interval [CI], 0.55–0.98). The multivariable‐adjusted model that included common prognostic factors as covariates showed that NAC‐DCF was associated with a significantly better prognosis than NAC‐CF (HR, 0.68; 95% CI, 0.52–0.90), consistent with the results of the IPW analysis (Table S1). Given the lower incidence of postoperative complications in the DCF group and their established prognostic relevance, we performed a sensitivity analysis restricted to patients who achieved R0 resection. The survival benefit associated with NAC‐DCF remained materially unchanged after additional adjustment for operative procedure and postoperative complications (HR, 0.57; 95% CI, 0.40–0.82; Table S2).

**FIGURE 3:**
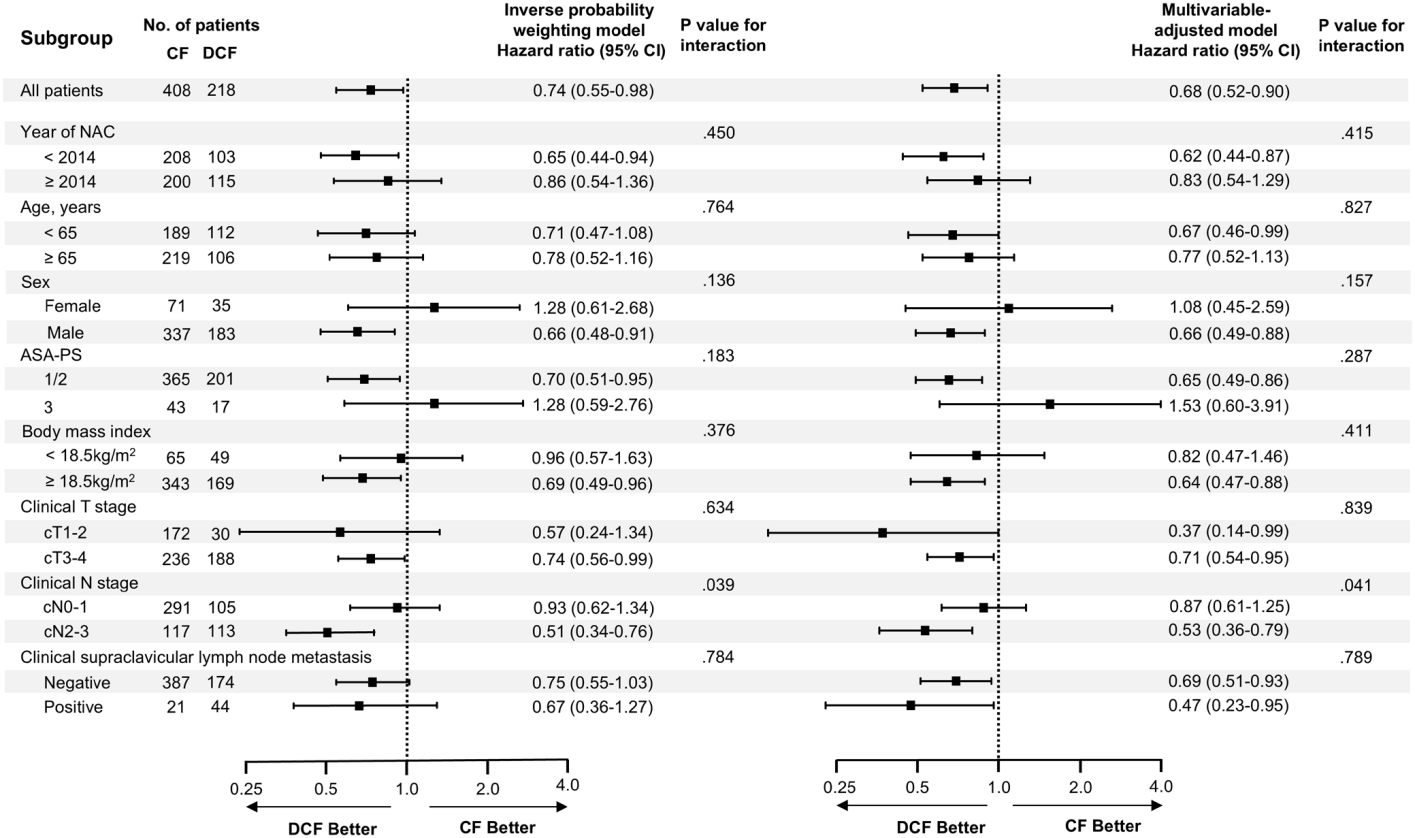
Forest plot of hazard ratios for overall survival, stratified by demographic and baseline disease characteristics, using both inverse probability weighting and a multivariable analysis model to evaluate the impact of NAC‐DCF versus NAC‐CF.

Subgroup analyses were then performed in both the IPW and the multivariable‐adjusted models. HRs for OS consistently favored the DCF group across demographic and baseline disease characteristics, with the exception of ASA‐PS 3 (Figure 3). Notably, among these characteristics, patients with cN2‐3 disease demonstrated a more favorable survival benefit with NAC‐DCF (HR, 0.51; 95% CI, 0.34–0.76 in the IPW analysis model) compared with patients with cN0‐1 disease (HR, 0.93; 95% CI, 0.62–1.34), and nominally significant interaction in the treatment effect of DCF was observed between these cN stages (*p* = 0.039 for the interaction). A similar trend was observed in the multivariable‐adjusted analysis.

### 3.5 OS And RFS in Patients With cN2‐3 Disease

**FIGURE 4:**
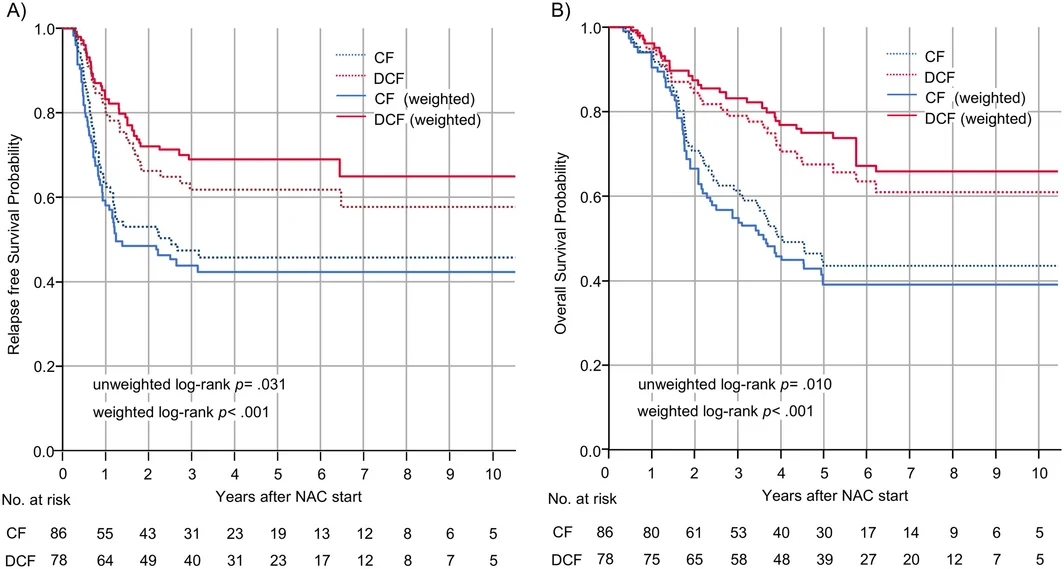
Kaplan–Meier estimates of crude and inverse probability weighting for relapse‐free survival (A) and overall survival (B) in patients with cN2‐3 between the NAC‐CF and NAC‐DCF groups.

Based on the previous subgroup analysis, a further examination of the data was conducted for those patients with cN2‐3 disease who underwent R0 resection. The DCF group with cN2‐3 disease exhibited more advanced cT and SCLNs metastases (Table S3). In the IPW model, individuals who received NAC‐DCF had a more markedly improved prognosis compared with those who received NAC‐CF, with a 5‐year RFS rate of 68.6% versus 41.2%, respectively (Figure 4A, wHR, 0.42; 95% CI, 0.25–0.69) and a 5‐year OS rate of 74.7% versus 38.6%, respectively (Figure 4B; wHR, 0.38; 95% CI, 0.23–0.63). A stratified analysis of patients with cT3N2–3 M0 disease also yielded comparable outcomes, with a 5 year OS rate of 71.4% for the NAC‐DCF group versus 42.6% for the NAC‐CF group (Figure S2; HR, 0.44; 95% CI, 0.24–0.82).

The most common recurrence pattern in patients with cN2‐3 disease was LN recurrence. The DCF group exhibited a lower overall recurrence rate than the CF group, especially a significantly reduced incidence of LN recurrence (29.5% versus 38.4%, respectively; Table 3, wHR, 0.37; 95% CI, 0.21–0.64 in the IPW model).

**TABLE 3:** Recurrence patterns after R0 resection by planned surgery in patients with cN2‐3 [Correction added on 2 May 2026, after first online publication: In Table 3, the alignment of columns “CF, DCF, IPW model, and Multivariable adjusted model” has been corrected.].

	CF (*n* = 86)		DCF (*n* = 78)		IPW model		Multivariable adjusted model	
					Weighted HR (95% CI)	*p* Value	HR (95% CI)	*p* Value
All recurrence	45	(52.3%)	30	(38.5%)	0.42 (0.25–0.69)	< 0.001	0.60 (0.38–0.96)	0.031
Locoregional^a^	4	(4.7%)	4	(5.1%)	1.21 (0.22–6.41)	0.822	1.81 (0.33–9.92)	0.493
Hematogenous^a^	13	(15.1%)	14	(18.0%)	0.66 (0.30–1.43)	0.290	0.94 (0.45–1.96)	0.860
Lymph node^a^	33	(38.4%)	23	(29.5%)	0.37 (0.21–0.64)	< 0.001	0.57 (0.34–0.97)	0.037
Disseminated^a^	3	(3.5%)	4	(5.1%)	0.79 (0.19–3.33)	0.898	0.93 (0.23–3.72)	0.917

Abbreviations: CF, fluorouracil and cisplatin; CI, confidence interval; DCF, fluorouracil, cisplatin, and docetaxel; HR, hazard ratio; IPW, inverse probability weighting.

^a^ There are overlapping patterns of recurrence.

## 4 Discussion

This study demonstrated that NAC‐DCF improves OS compared with NAC‐CF in patients with resectable advanced ESCC. The pathological complete response (pCR) rate was also substantially higher in the DCF group (16.8%) than in the CF group (6.8%), and short‐term surgical outcomes were feasible in both groups. These results confirm the findings of the JCOG1109 trial, even in a large single‐institute, real‐world cohort. Among the results, a clinically important and novel observation is that the survival benefit of NAC‐DCF was more pronounced in patients with cN2‐3 LN metastases. Additionally, among the postoperative recurrence patterns in these patients, LN recurrence was significantly reduced. These findings may suggest that DCF could help control extensive lymph node metastasis and that its therapeutic effect might be observed more readily in the setting of more advanced nodal disease.

Although this pattern may appear to differ from the trend suggested by the cN‐stratified subgroup analysis in JCOG1109, these differences may be explained, at least in part, by the baseline distribution of occult poor prognostic factors—such as borderline resectability, suspicion of extranodal extension or adjacent organ invasion by nodal metastases or greater tumor burden—and indication‐driven treatment selection inherent to observational research. Accordingly, even among patients classified as cN0–1, those selected for intensive DCF through multidisciplinary discussion may have had a higher prevalence of clinically challenging features not fully captured by the TNM staging system used in this study, thereby potentially attenuating the observable survival benefit associated with DCF.

In contrast, in the cN2–3 subgroup—clinically indicative of more advanced nodal disease—the biological effect of DCF may have been sufficiently pronounced to overcome potential selection biases, thereby allowing its therapeutic benefit to be detected more readily. Importantly, these findings are not intended to challenge the randomized evidence established by JCOG1109, but rather to complement it by providing insight into how treatment effects may manifest in real‐world clinical practice. Therefore, the findings of this study—particularly those regarding the efficacy of DCF in patients with cN0–1 disease—should be interpreted with appropriate caution, taking the potential influence of selection bias into consideration.

Postoperative complications were less frequent in the NAC‐DCF group, likely reflecting greater physiological reserve among patients selected for intensive therapy. Given that pneumonia and anastomotic leakage substantially influence prognosis [12], we adjusted for these events in a multivariable model. The survival benefit of NAC‐DCF remained unchanged, indicating that differences in complication rates do not explain the primary findings.

Preoperative DCF trials were previously designed as induction therapy with the intent of achieving R0 resection in locally advanced disease at the margin of resectability [13, 14]. However, this study shifts the focus to resectable disease with multiple LN metastases. Notably, this survival advantage persisted even after restricting the cohort to patients who achieved R0 resection, suggesting that the benefit of DCF is not merely attributable to improved resectability. In advanced esophageal adenocarcinoma, novel docetaxel‐based NAC regimens, such as DCF and FLOT, have been reported to be effective in sterilizing metastatic LNs in addition to shrinking the primary tumor compared with conventional regimens [15]. Regarding ESCC trials, in a phase II clinical trial of DCF for ESCC with distant metastases, DCF was particularly effective against lymphatic metastases, whereas its efficacy against hematogenous metastases appeared to be more limited [16]. This finding was consistent with the recurrence pattern observed in this study, in which LN recurrences were more effectively suppressed in the DCF group compared with the CF group, while hematogenous recurrence also showed a numerically favorable trend without reaching statistical significance. DCF may exert systemic effects as a triplet chemotherapy regimen. In the present study, DCF contributed to improved disease control in tumors characterized by advanced lymphatic invasion; however, no clear effect on hematogenous recurrence was observed, possibly reflecting the limited number of events.

A subgroup analysis of the CROSS trial [17] suggested limited neoadjuvant CRT efficacy in LN‐positive patients. The 10 year outcomes indicated that neoadjuvant CRT reduced locoregional recurrence but lacked impact on systemic relapse [18]. The JCOG1109 trial showed that postoperative distant metastases were better controlled in the NAC‐DCF group than in the neoadjuvant CRT group. These findings suggested that in the United States and Europe where neoadjuvant CRT is the standard treatment, intensified systemic therapy, such as NAC‐DCF or NAC‐FLOT, may be a viable alternative for ESCC with multiple LN metastases rather than for locally advanced disease, with the goal of improving distant metastasis control. For patients at high risk of distant recurrence, immune checkpoint inhibitor therapy may be considered in addition to preoperative therapy; the results of the CheckMate 577 trial showed that patients who received nivolumab as postoperative therapy after neoadjuvant CRT had longer disease‐free survival compared with the placebo group [19]. It may be noted, however, that patients with esophageal cancer are generally in poor condition and may refuse postoperative therapy.

Considering the frequency of FN associated with DCF, it is crucial to select an appropriate NAC regimen based on the capacity of the treatment institute, and consideration of patient characteristics is crucial to avoid unnecessary adverse events. In a stratified analysis based on patient characteristics in the JCOG1109 trial, no specific characteristic showed a difference in treatment efficacy. However, a reevaluation using real‐world clinical practice data showed that NAC‐DCF was particularly beneficial compared with NAC‐CF for patients with resectable advanced ESCC with cStage III and IVA, while it was not beneficial for elderly patients aged ≥ 75 years [9]. In our study, although patients over 75 years of age were not included, patients with ASA‐PS 3 tended to show less benefit. Therefore, incorporating the results of this study, a doublet NAC‐CF regimen may be proposed as a treatment option for elderly patients or those with severe comorbidities, if NAC is indicated but they have few LN metastases or early‐stage tumors.

Preoperative diagnosis of LN metastases is crucial for determining treatment strategies based on the cN stage. Diagnosing LN metastasis in esophageal cancer can be challenging due to the diversity of LN size, skip metastases via the submucosal lymphatic system, and the need to evaluate nodes from the neck to the abdomen. Clinical diagnosis relies primarily on LN size, morphology, and contrast enhancement on CT, with FDG‐PET as an adjunct [20]; however, criteria vary by LN location and require expertise. In this single‐institute study, diagnoses were made using uniform criteria through a multidisciplinary team conference led by esophageal cancer experts, ensuring diagnostic consistency—a strength not present in multicenter trials or national database studies. However, with respect to the accuracy of our LN diagnostic criteria, diagnostic performance remains suboptimal, although they are based on CT and PET/CT assessments and incorporate LN size and configuration as well as region‐specific parameters, representing the most practical and widely reported approach [21, 22, 23]. Further research is needed to develop imaging diagnostic systems using innovative techniques, such as deep learning with artificial intelligence [24].

Some limitations of our study should be noted. First, it was a retrospective study, and selection bias cannot be completely eliminated. We have made efforts to address this limitation by applying IPW and multivariable‐adjusted models. The consistent findings across both models suggest that the influence of selection bias was minimized. Second, the subgroup analysis aimed to identify tumor characteristics most favorable for NAC‐DCF; however, the sample sizes within each subgroup were limited. Nevertheless, despite being conducted at a single institution, a total of 626 patients who received NAC were enrolled, including 230 patients with cN2–3 disease. Furthermore, with a median follow‐up of 70.1 months for survivors, we believe that sufficient data are available for analysis. Third, adverse event profiling during NAC was not feasible due to inconsistent and incomplete documentation in the clinical records. However, we have reported previously on our institute's preoperative DCF experience and the associated adverse events [7, 8], which were found to be acceptable in terms of safety.

In conclusion, NAC‐DCF provided better OS compared with NAC‐CF in resectable advanced ESCC, with a higher pCR rate and feasible surgical outcomes. Subgroup analyses suggested a potential benefit in patients with cN2–3 disease, accompanied by reduced lymph node recurrence. Although cautious interpretation is warranted given the retrospective design, these findings may represent hypothesis‐generating observations derived from real‐world clinical practice and may help inform the development of individualized treatment strategies that balance therapeutic efficacy with toxicity risk.

## Author Contributions

**Eiji Higaki:** conceptualization, methodology, resources, data curation, investigation, visualization, writing – original draft, writing – review and editing. **Shigenori Kadowaki:** methodology, resources, validation, writing – review and editing. **Tetsuya Abe:** supervision, resources, writing – review and editing. **Masahiro Tajika:** resources, writing – review and editing. **Tsutomu Tanaka:** resources, writing – review and editing. **Hironori Fujieda:** resources, writing – review and editing, data curation, investigation. **Koji Komori:** writing – review and editing. **Seiji Ito:** writing – review and editing. **Isao Oze:** supervision, methodology, validation, visualization, writing – review and editing. **Kei Muro:** supervision, resources, writing – review and editing.

## Funding

The authors have nothing to report.

## Ethics Statement

This study was approved by the Institutional Review Board of the Aichi Cancer Center Hospital (Approval No. ACC 2021–1‐043). Owing to the retrospective design of the study, informed consent was obtained through an opt‐out process.

## Conflicts of Interest

The authors declare no conflicts of interest.

## Supporting information


**Figure S1:** (a) Density distribution of propensity scores in the CF and DCF groups, (b) Standardized mean difference (SMD) of confounding variables.

**Figure S2:** Kaplan–Meier estimates of relapse‐free survival and overall survival in patients with cT3N2–3 M0 disease who underwent R0 resection.

**Table S1:** Cox regression analysis of hazard ratios for overall survival.

**Table S2:** Cox regression analysis of overall survival in patients who achieved R0 resection.

**Table S3:** Pre‐treatment baseline characteristics of patients diagnosed with cN2‐3 and undergoing R0 resection by planned surgery.

## Data Availability

The data that support the findings of this study are available from the corresponding author upon reasonable request.
