# Supplementary material for: Potential Survival Benefit of Neoadjuvant Docetaxel, Cisplatin and 5‐Fluorouracil Therapy in Patients With Esophageal Squamous Cell Carcinoma With Multiple Lymph Node Metastases: A Single‐Institute Propensity Score Analysis
Source: Ann Gastroenterol Surg. 2026 Apr 19;10(5):1496–506. doi: 10.1002/ags3.70224 (PMC13394455; doi:10.1002/ags3.70224)
Supplement: Supplementary file 1 [file AGS3-10-1496-s001.docx]

**Supplementary Table S1.** Cox regression analysis of hazard ratios for overall survival

| Variable | **n** | **HR (95% CI)** | ***P value*** |
| --- | --- | --- | --- |
| Year of NAC start |  |  | *.004* |
| < 2014 | 311 | Reference |  |
| ≥ 2014 | 315 | 0.69 (0.53-0.89) |  |
| Age |  |  | *.516* |
| < 65 yr | 301 | Reference |  |
| ≥ 65 yr | 325 | 1.09 (0.85-1.39) |  |
| Gender |  |  | *.002* |
| Female | 106 | Reference |  |
| Male | 520 | 1.83 (1.25-2.68) |  |
| ASA-PS |  |  | *.001* |
| 1-2 | 566 | Reference |  |
| 3 | 60 | 1.83 (1.26-2.64) |  |
| BMI |  |  | *.001* |
| ≥ 18.5 kg/m^2^ | 114 | Reference |  |
| < 18.5 kg/m^2^ | 512 | 1.69 (1.25-2.28) |  |
| Clinical T stage† |  |  | *<.001* |
| cT1-2 | 202 | Reference |  |
| cT3-4 | 424 | 2.79 (1.99-3.90) |  |
| Clinical N stage† |  |  | *.011* |
| cN0-1 | 396 | Reference |  |
| cN2-3 | 230 | 1.41 (1.08-1.83) |  |
| Clinical supraclavicular lymph node metastasis | | | *.007* |
| Negative | 561 | Reference |  |
| Positive | 65 | 1.61 (1.13-2.27) |  |
| NAC regimen |  |  | *.006* |
| CF | 408 | Reference |  |
| DCF | 218 | 0.68 (0.52-0.90) |  |
| Abbreviations: HR, hazard ratio; CI, confidence interval; NAC, neoadjuvant chemotherapy; ASA-PS, American Society of Anesthesiologists physical status; BMI, body mass index; CF, fluorouracil and cisplatin; DCF, fluorouracil, cisplatin, and docetaxel  †TNM classification of International Union Against Cancer (UICC) 8th edition. | | | |
