# Supplementary material for: Potential Survival Benefit of Neoadjuvant Docetaxel, Cisplatin and 5‐Fluorouracil Therapy in Patients With Esophageal Squamous Cell Carcinoma With Multiple Lymph Node Metastases: A Single‐Institute Propensity Score Analysis
Source: Ann Gastroenterol Surg. 2026 Apr 19;10(5):1496–506. doi: 10.1002/ags3.70224 (PMC13394455; doi:10.1002/ags3.70224)
Supplement: Supplementary file 3 [file AGS3-10-1496-s004.docx]

**Supplementary Table S3.** Pre-treatment baseline characteristics of patients diagnosed with cN2-3 and undergoing R0 resection by planned surgery

| **Variable** | **CF (n=86)** | | **DCF (n=78)** | | ***P Value*** | |
| --- | --- | --- | --- | --- | --- | --- |
| Year of NAC start ≥2014 | 47 | (54.7%) | 48 | (61.5%) | *.372* | |
| Age, median (IQR), yr | 66 | (60-70) | 63 | (58-68) | *.057* | |
| Male, n (%) | 72 | (83.7%) | 65 | (83.3%) | *.947* | |
| BMI median (IQR), kg/m^2^ | 21.8 | (19.6-23.5) | 21.7 | (19.1-23.6) | *.674* | |
| ASA-PS, n (%) |  | | | | *.633* |  |
| 1 | 26 | (30.2%) | 29 | (37.2%) |  | |
| 2 | 52 | (60.5%) | 43 | (55.1%) |  |  |
| 3 | 8 | (9.3%) | 6 | (7.7%) |  |  |
| Diabetes mellitus, n (%) | 9 | (10.5%) | 6 | (7.7%) | *.538* | |
| Cardiovascular disease, n (%) | 43 | (50.0%) | 30 | (38.5%) | *.138* | |
| FEV_1.0_%, median (IQR), % | 76.8 | (73.7-82.0) | 75.7 | (71.0-80.0) | *.674* | |
| Brinkman index, median (IQR) | 640 | (360-1020) | 627 | (360-1000) | *.711* | |
| Tumor location |  | | | | *.169* |  |
| Proximal third | 7 | (8.1%) | 14 | (18.0%) |  | |
| Middle third | 42 | (48.8%) | 35 | (44.9%) |  |  |
| Distal third | 37 | (43.0%) | 29 | (37.2%) |  |  |
| Clinical T stage† |  | | | | *.017* |  |
| 1 | 10 | (11.6%) | 5 | (6.4%) |  | |
| 2 | 11 | (12.8%) | 1 | (1.3%) |  |  |
| 3 | 64 | (74.4%) | 70 | (89.7%) |  |  |
| 4a | 1 | (1.2%) | 2 | (2.6%) |  |  |
| Clinical N stage† |  |  |  |  | *.453* | |
| 2 | 80 | (93.0%) | 70 | (89.7%) |  | |
| 3 | 6 | (7.0%) | 8 | (10.3%) |  |  |
| Supraclavicular lymph node metastasis | 6 | (7.0%) | 22 | (28.2%) | *<.001* | |
| Abbreviations: CF, fluorouracil and cisplatin; DCF, fluorouracil, cisplatin, and docetaxel; NAC, neoadjuvant chemotherapy; IQR, interquartile range; BMI, body mass index; ASA-PS, American Society of Anesthesiologists Physical Status  †TNM classification of International Union Against Cancer (UICC) 8th edition. | | | | | | |
