# Supplementary figures and images for: Potential Survival Benefit of Neoadjuvant Docetaxel, Cisplatin and 5‐Fluorouracil Therapy in Patients With Esophageal Squamous Cell Carcinoma With Multiple Lymph Node Metastases: A Single‐Institute Propensity Score Analysis
Source: Ann Gastroenterol Surg. 2026 Apr 19:10.1002/ags3.70224. Online ahead of print. doi: 10.1002/ags3.70224 (PMC13394455; doi:10.1002/ags3.70224)

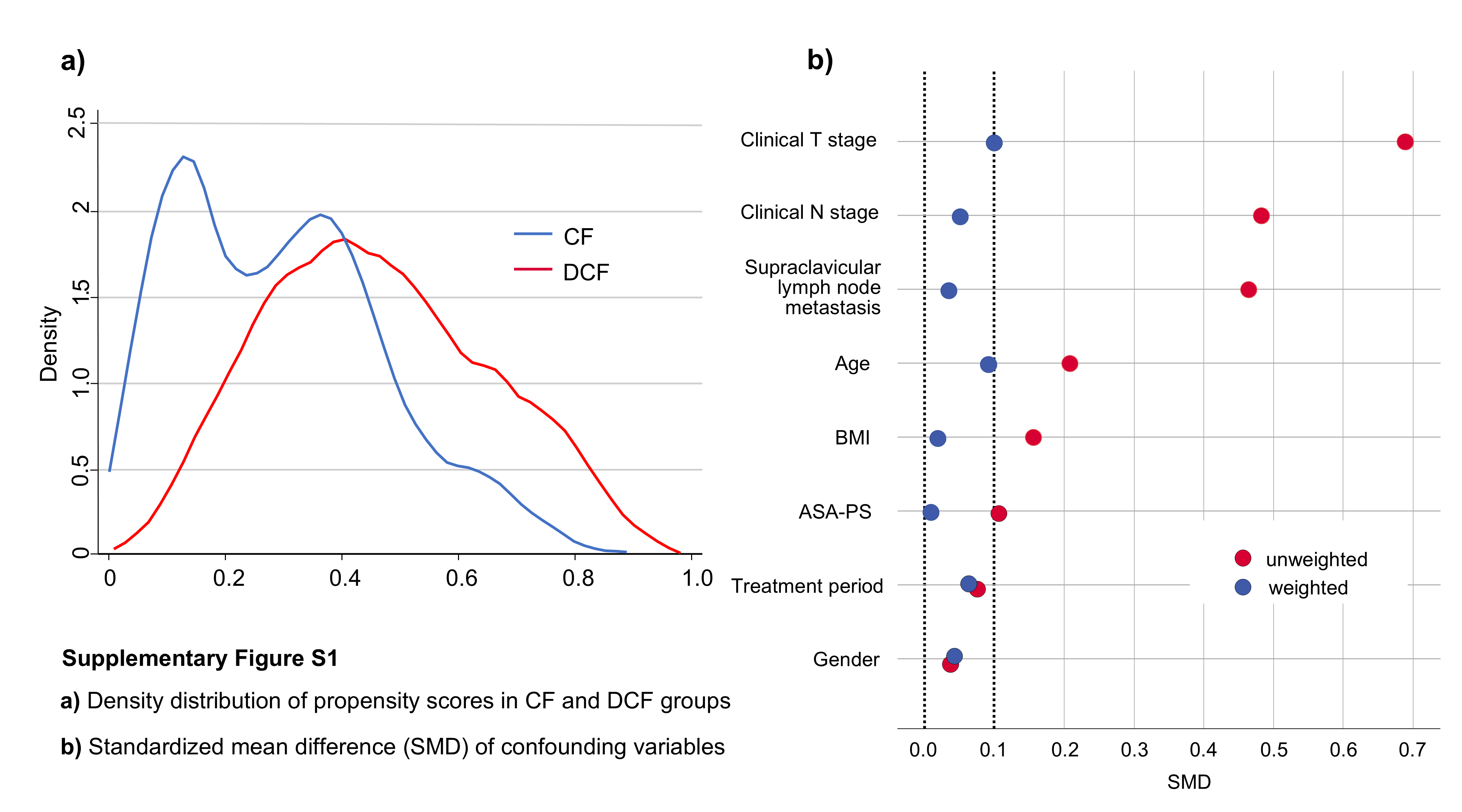

Supplement: Supplementary file 1 — Figure S1: (a) Density distribution of propensity scores in the CF and DCF groups, (b) Standardized mean difference (SMD) of confounding variables. [file AGS3-9999-0-s002.tif]

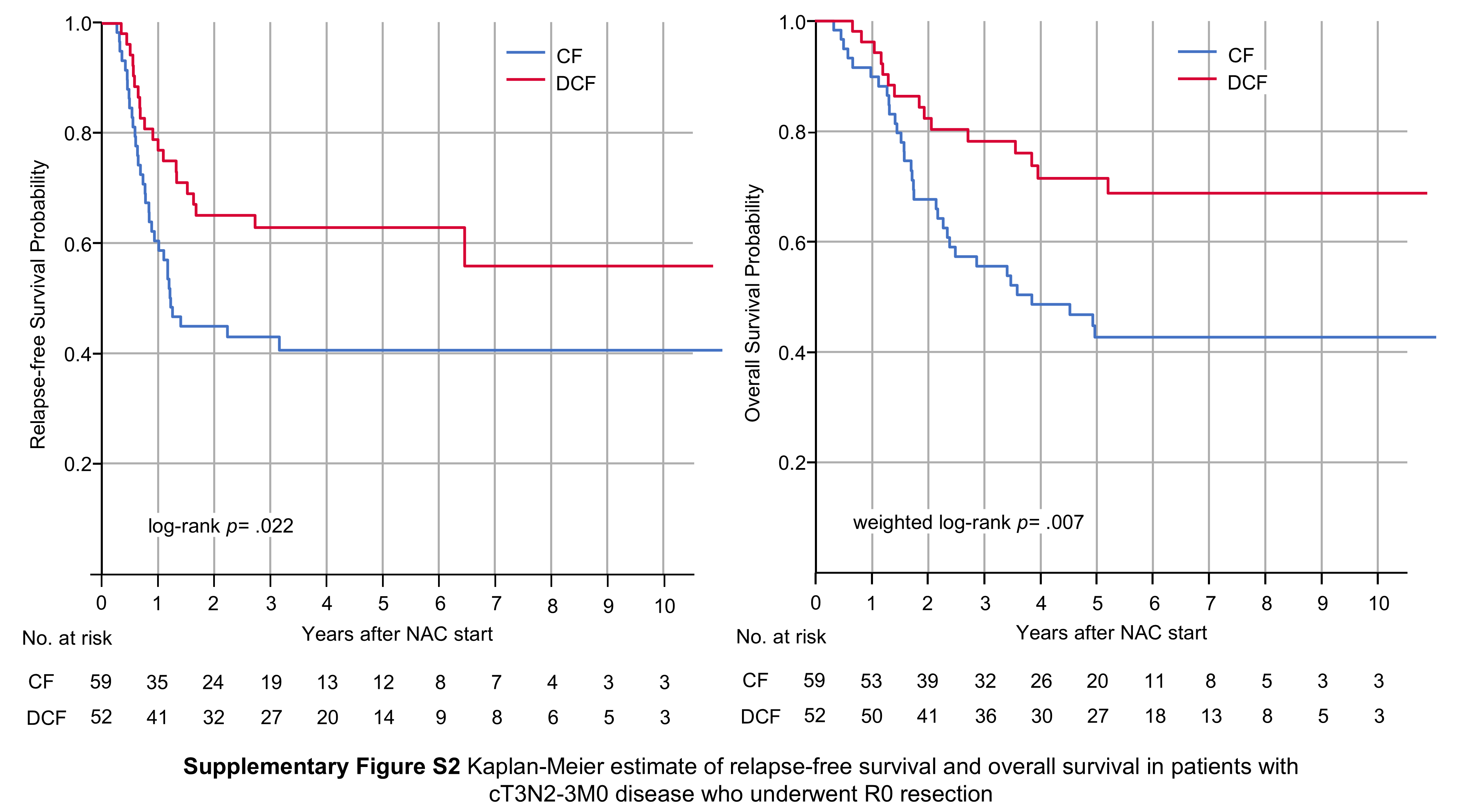

Supplement: Supplementary file 2 — Figure S2: Kaplan–Meier estimates of relapse‐free survival and overall survival in patients with cT3N2–3 M0 disease who underwent R0 resection. [file AGS3-9999-0-s005.tif]
