# Supplementary material for: Potential Survival Benefit of Neoadjuvant Docetaxel, Cisplatin and 5‐Fluorouracil Therapy in Patients With Esophageal Squamous Cell Carcinoma With Multiple Lymph Node Metastases: A Single‐Institute Propensity Score Analysis
Source: Ann Gastroenterol Surg. 2026 Apr 19:10.1002/ags3.70224. Online ahead of print. doi: 10.1002/ags3.70224 (PMC13394455; doi:10.1002/ags3.70224)
Supplement: Supplementary file 4 — Table S2: Cox regression analysis of overall survival in patients who achieved R0 resection. [file AGS3-9999-0-s003.docx]

**Supplementary Table S2.** Cox regression analysis of overall survival in patients who achieved R0 resection

| **Variable** | **n** | **HR (95% CI)** | ***P value*** |
| --- | --- | --- | --- |
| Year of NAC start | |  | *.034* |
| < 2014 | 234 | Reference |  |
| ≥ 2014 | 254 | 0.59 (0.36-0.96) |  |
| Age |  |  | *.181* |
| < 65 yr | 238 | Reference |  |
| ≥ 65 yr | 250 | 1.23 (0.91-1.68) |  |
| Gender |  |  | *.028* |
| Female | 89 | Reference |  |
| Male | 399 | 1.68 (1.06-2.65) |  |
| ASA-PS |  |  | *.046* |
| 1-2 | 444 | Reference |  |
| 3 | 44 | 1.63 (1.01-2.63) |  |
| BMI |  |  | *.001* |
| ≥ 18.5 kg/m^2^ | 399 | Reference |  |
| < 18.5 kg/m^2^ | 89 | 1.92 (1.31-2.81) |  |
| Clinical T stage† |  |  | *<.001* |
| cT1-2 | 179 | Reference |  |
| cT3-4 | 309 | 2.55 (1.71-3.80) |  |
| Clinical N stage† |  |  | *.003* |
| cN0-1 | 324 | Reference |  |
| cN2-3 | 164 | 1.64 (1.18-2.29) |  |
| Clinical supraclavicular lymph node metastasis | | | *.017* |
| Negative | 441 | Reference |  |
| Positive | 47 | 1.73 (1.10-2.72) |  |
| Operative procedure |  |  |  |
| Transthoracic esophagectomy | 244 | Reference |  |
| Thoracoscopic esophagectomy | 241 | 0.96 (0.58-1.58) | *.868* |
| Transhiatal esophagectomy | 3 | 0.94 (0.12-7.35) | *.955* |
| Postoperative pneumonia |  |  | *.110* |
| No | 379 | Reference |  |
| Yes | 109 | 1.33 (0.94-1.89) |  |
| Anastomotic leakage | | | *.158* |
| No | 430 | Reference |  |
| Yes | 58 | 1.36 (0.89-2.07) |  |
| Recurrent laryngeal nerve palsy | | | *.401* |
| No | 382 | Reference |  |
| Yes | 106 | 0.85 (0.59-1.24) |  |
| NAC regimen |  |  | *.002* |
| CF | 330 | Reference |  |
| DCF | 158 | 0.57 (0.40-0.82) |  |
| Abbreviations: HR, hazard ratio; CI, confidence interval; NAC, neoadjuvant chemotherapy; ASA-PS, American Society of Anesthesiologists physical status; BMI, body mass index; CF, fluorouracil and cisplatin; DCF, fluorouracil, cisplatin, and docetaxel;  †TNM classification of International Union Against Cancer (UICC) 8th edition. | | | |
